# Use of Antibiotics in Pediatrics: 8-Years Survey in Italian Hospitals

**DOI:** 10.1371/journal.pone.0139097

**Published:** 2015-09-25

**Authors:** Elena Buccellato, Mauro Melis, Chiara Biagi, Monia Donati, Domenico Motola, Alberto Vaccheri

**Affiliations:** Unit of Pharmacology, Department of Medical and Surgical Sciences, University of Bologna, Via Irnerio 48, 40126, Bologna, Italy; Aligarh Muslim University, INDIA

## Abstract

**Objectives:**

To evaluate antibiotic consumption in the pediatric wards of Emilia-Romagna Region, from 2004 to 2011, with a focus on the antibiotics reserved to the most serious infections, and to analyse the ADRs reported for antibiotics by the pediatric wards of Emilia-Romagna hospitals.

**Methods:**

Reference population was represented by all the patients (0–14 years old) admitted to the pediatric wards of all the hospitals of Emilia-Romagna Region. Drug consumption was expressed as number of DDDs per 100 Bed-Days (BD) and data were analysed by active substance, by therapeutic subgroups or by ward type. The time trends of antibiotic consumption were statistically analysed by linear regression. All the suspected ADR reports associated with antibiotics, reported between January 2004 and December 2011 were drawn by the Italian Spontaneous Reporting Database.

**Results:**

Overall antibiotic consumption showed only a slight increase (p = 0.224). Among the pediatric wards, pediatric surgery showed the highest increase from 2004 to 2011 (p = 0.011). Penicillins and β-lactamase inhibitors was the first therapeutic group with a statistically significant increase over years (p = 0.038), whereas penicillins with extended spectrum presented a statistically significant reduction (p = 0.008). Moreover, only 5 drugs out of the 8 antibiotics reserved to the most serious infections were used. Pharmacovigilance data showed 27 spontaneous ADR reports associated to ATC J01 drugs. Amoxicillin/clavulanic acid had the highest number of ADR reports (n = 7).

**Conclusions:**

The steadily increasing consumption in penicillins and β-lactamase inhibitors, in association with a considerable decrease of plain penicillins, raises a serious concern. Pharmacovigilance reports seem to suggest a safe use of antibiotics in the hospital setting of Emilia-Romagna. Further studies to investigate the reason for prescribing antibiotics in children inpatients are needed.

## Introduction

Antibiotics are widely prescribed therapeutic agents[1] and their use in the pediatric population is worryingly high in some countries.[2–4] It is well known that a considerable amount of antibiotics is prescribed to children for conditions such as upper respiratory tract infections, which are often caused by viral agents and easily self-managed.[5,6] The reduction of unnecessary antibiotic use is one of the main goals of healthcare authorities to reduce the occurrence of antibiotic resistance, caused by overuse/misuse of these drugs.[7–12]

A wide variability in antibiotic consumption in outpatient pediatric population exists between countries.[13] In 2011, Italy ranked 9^th^ on total antibiotic use among 41 states according to the report in the WHO (World Health Organization) European Region.[14] It is well documented that an Italian child has an higher risk of being exposed to antibiotics than an English or Dutch or Danish child,[13,15,16] and many qualitative differences are already known: the second line treatments (e.g. third-generation cephalosporins) in most infectious diseases are very common in Italy, whereas they are only seldom prescribed in the Northern European countries.[16] Substantial differences have also been reported in Italy between Northern and Southern regions.[17]

Exposure of children to bacterial infections is higher than in adults, because the immune system is not fully developed in childhood and children tend to be exposed to more disease-causing bacteria through day-to-day activities such as childcare and mouthing behaviours, especially among infants.[18] Treatment options are limited in childhood, but an inappropriate use of antibacterial agents may promote the emergence of antibiotic-resistant bacteria. Population exposure to antibiotics is recognised as a cause for the growing of resistance to common antibacterial agents among a majority of bacterial strains. More often bacteria are exposed to antibiotics, more resistant they become.[18]

In addition, the interest about antibacterial agents has strongly increased in the last years since some antibiotic groups were associated with cardiovascular risks.[19–24] Thus, increased consumption of antibiotics requires monitoring of adverse drug reactions (ADRs), in order to assess the risk-benefit ratio of marketed drugs. The spontaneous reporting system (SRS) is one the most effective methods for the detection of drug reactions: the assessment of antibiotics use with the related ADRs can provide an additional perspective to healthcare professionals.

Although a few studies have been performed to investigate the profile of drug use and the ADRs frequency in Italian outpatient children, [3,13,15,16,25–31] limited data are available for the hospital setting.[32,33]

As reported in a previous paper of ours,[34] the overall antibiotic use from 2004 to 2011 in all pediatric wards grew in Emilia-Romagna hospitals, in spite of a decrease in expenditure likely because of a positive influence of generics on drug prices.

The aim of the present study is to evaluate the trends of antibiotic consumption in the pediatric wards of all Emilia-Romagna Region hospitals, Italy, from 2004 to 2011, with special regard to those drugs that should be reserved to the most serious infections. Moreover, the ADRs reported for antibiotics by the pediatric wards of Emilia-Romagna hospitals will be analysed.

## Methods

### Study population

Reference population was represented by all the patients (0–14 years old) admitted to the pediatric wards of all the hospitals of Emilia-Romagna Region. People aged 15 years and older were not included in the analysis. Data concerning hospital discharge or outpatient visits were not included as well.

The different pediatric specialties existing in the various hospitals have been attributed to four ward types: neonatology, pediatrics, pediatric onchoematology and pediatric surgery. The data from day hospital facilities, both in terms of drug consumption and of bed-days, were added to those of the corresponding wards. The number of bed-days for the individual pediatric wards within each hospital was obtained from the Emilia-Romagna Region hospital discharge database (Table 1).

**Table 1:** List of the hospitals and pediatric wards of Emilia-Romagna Region included in the analysis. Figures represent the number of bed-days in 2011.

Hospitals	Wards			
	Pediatric surgery	Neonatology	Pediatrics	Onchoematology
GH Piacenza	-	8340	6200	-
GH Parma	-	2742	150	-
GH Reggio Emilia	-	7058	2190	-
GH Modena	-	9162	7461	-
GH Bologna	1661	14640	11964	-
GH Imola	-	2701	4020	-
GH Ferrara	-	3762	1914	-
GH Ravenna	-	11589	5395	-
GH Forlì	-	4470	2138	-
GH Cesena	-	7760	5530	-
GH Rimini	1877	11865	10629	-
UH Parma	3269	11554	8830	7565
UH Reggio Emilia	-	11800	8973	-
UH Modena	2210	15532	7544	3691
UH Bologna	11303	14945	18094	7892
UH Ferrara	2485	7472	13142	-

GH = General Hospital

UH = University Hospital

### Drug utilization data

Data of antibacterial agents consumption (ATC group J01) in the period 2004 to 2011 were obtained from the Emilia-Romagna Health Authority Database, which collects the pharmacy records of each hospital. The database provides the following information for each drug dispensed: identification of the product according to the Anatomical Therapeutic Chemical (ATC) classification, number of dose units and number of defined daily doses (DDDs) dispensed, code of the ward, and year of the prescription. ATC and DDD referred to the international classification published by the WHO Collaborating Centre for Drug Statistics Methodology, 2012 edition. Drug consumption was expressed as number of DDDs per 100 Bed-Days (BD) and data were analysed by active substance (ATC 5th level), by therapeutic subgroups (ATC 4th level), or by ward type.

We also analysed the use of those antibiotics (tigecycline, ertapenem, ceftaroline, telithromycin, quinupristin/daflopristin, teicoplanin, linezolid, daptomycin) that can be used only after a patient-specific request to the hospital pharmacy, as required by the Emilia-Romagna Region Hospital Formulary.

The time trends of antibiotic consumption were statistically analysed by linear regression.

### Pharmacovigilance data

All the suspected ADR reports associated with antibiotics (ATC J01), reported between January 2004 and December 2011 were drawn by VigiSegn, the Italian Spontaneous Reporting Database. The ADRs reported by Emilia-Romagna hospitals were collected by age of the patients (pediatric age classes) and source of reporting (hospital or specialist doctor and nurse). Reports from literature have been excluded from the analysis.

## Results

### Drug utilization analysis

In 2011 the pediatric population of Emilia-Romagna accounted for 582,105, and increased by 25.2% from 2001.[35] A total of 87,970 pediatric inpatients were recorded in the wards selected for the present study of Emilia-Romagna hospitals in 2011.

Overall antibiotic consumption showed a slight and not significant increase from 35.60 DDD/100 BD in 2004 to 42.39 DDD/100 BD in 2011 (b = 0.420; IC95% −0.339–1.180; p = 0.224) (Table 2).

**Table 2:** Trend of antibiotic consumption in the pediatric wards of Emilia-Romagna hospitals from 2004 to 2011.

Wards	DDD/100 BD by year								Trend analysis			
	2004	2005	2006	2007	2008	2009	2010	2011	b	95% Lower limit	95% Upper limit	P
Pediatric surgery	56.07	80.39	79.22	83.73	81.94	92.45	89.32	91.12	3.904	1.273	6.535	0.011
Neonatology	7.42	6.64	6.41	8.36	7.13	7.69	8.04	8.58	0.211	-0.029	0.452	0.075
Pediatrics	63.56	73.30	75.85	74.00	67.62	71.09	69.93	76.88	0.663	-1.023	2.349	0.373
Pediatric onchoematology	49.63	34.74	34.70	35.00	35.75	34.40	34.62	35.37	-1.198	-2.955	0.559	0.146
**TOTAL**	**35.60**	**40.16**	**39.56**	**39.60**	**36.43**	**38.65**	**38.90**	**42.39**	**0.420**	**-0.339**	**1.180**	**0.224**

BD = Bed-Days

Among the pediatric wards of Emilia-Romagna hospitals, pediatric surgery showed the highest antibiotic consumption in 2011 (91.12 DDD/100 BD), followed by paediatrics and pediatric onchoematology, whereas neonatology had a much lower consumption. Pediatric surgery also showed the highest increase from 2004 to 2011 (b = 3.904; IC95% 1.273–6.535; p = 0.011); no statistically significant trends were observed for neonatology and pediatrics, whereas a non significant decrease of antibiotic consumption was observed in pediatric onchoematology (Table 2).

Oral administration was the most common route of administration (53.67%), whereas parenteral drugs showed a slightly lower frequency (46.32%). The remaining 0.01% accounted for inhaled products.

Table 3 shows the ranking of ATC groups (ATC 4th level) during the period 2004–2011. Among the 26 therapeutic subgroups identified, penicillins and β-lactamase inhibitors (ATC J01CR) had the highest frequency both in 2004 and 2011 (10.91 DDD/100 BD, 30.6%; 15.22 DDD/100 BD, 35.9%, respectively), with a statistically significant increase (b = 0.362; IC95% 0.028–0.696; p = 0.038). J01CR was the first therapeutic group in paediatrics with 26.90 DDD/100 BD in 2011, pediatric onchoematology (9.37 DDD/100 BD) and pediatric surgery (46.57 DDD/100 BD). Amoxicillin/clavulanic acid was the most consumed drug representing about one-fourth (26.3%) of all antibiotics in 2011, whereas plain amoxicillin was the 5th drug with the 6.4% of the total amount in 2011.[S1 Table] Neonatology mostly used J01CA group (penicillins with extended spectrum), followed by J01CR (2.73 DDD/100 BD and 1.91 DDD/100 BD in 2011, respectively).

**Table 3:** Trend of ATC 4^th^ groups from 2004 to 2011 in pediatric inpatients in Emilia-Romagna hospitals. Groups are ranked by decreasing consumption in 2011.

ATC IV	Antimicrobial group	DDD/100 BD								Trend analysis			
		2004	2005	2006	2007	2008	2009	2010	2011	b	95% Lower limit	95% Upper limit	P
J01CR	Penicillins + Beta-lactamase inhibitors	10.91	13.63	13.85	13.70	13.12	13.74	13.86	15.22	0.362	0.028	0.696	0.038
J01DD	Third-generation cephalosporins	6.00	6.61	6.30	6.31	5.84	6.61	6.59	6.90	0.080	-0.039	0.199	0.153
J01FA	Macrolides	4.26	4.78	4.53	4.81	4.04	4.91	5.31	5.67	0.154	0.000	0.307	0.050
J01CA	Penicillins with extended spectrum	5.85	6.10	5.31	5.67	5.03	5.11	4.83	5.06	-0.156	-0.254	-0.058	0.008
J01GB	Aminoglycosides	2.66	2.44	2.85	2.77	2.34	2.18	2.38	2.37	-0.057	-0.134	0.019	0.117
J01DC	Second-generation cephalosporins	1.17	1.43	1.42	1.29	1.16	0.83	1.07	1.24	-0.039	-0.107	0.030	0.220
J01DH	Carbapenems	0.65	0.89	1.00	0.74	0.83	0.97	0.53	0.93	0.002	-0.065	0.069	0.947
J01XA	Glycopeptides	0.81	0.78	0.80	0.89	0.80	0.98	0.89	0.91	0.021	0.000	0.041	0.048
J01XD	Imidazole derivatives	0.28	0.45	0.72	0.48	0.61	0.66	0.63	0.83	0.055	0.012	0.098	0.020
J01MA	Fluoroquinolones	0.71	0.71	0.72	0.74	0.70	0.56	0.64	0.80	-0.003	-0.031	0.026	0.838
J01EE	Combinations of sulfonamides and trimethoprim	1.11	0.94	0.91	0.67	0.69	0.46	0.59	0.72	-0.068	-0.120	-0.017	0.018
J01EC	Intermediate-acting sulfonamides	0.26	0.18	0.21	0.45	0.07	0.42	0.42	0.37	0.026	-0.024	0.076	0.250
J01DB	First-generation cephalosporins	0.11	0.13	0.31	0.36	0.38	0.34	0.38	0.33	0.035	0.006	0.063	0.025
J01XX	Other antibacterials	0.11	0.11	0.03	0.03	0.09	0.14	0.24	0.27	0.025	0.000	0.050	0.053
	All other antibacterials	0,72	0,97	0,61	0,69	0,72	0,74	0,55	0,77	-0.016	-0.063	0.032	0.451
	**TOTAL**	**35.60**	**40.16**	**39.56**	**39.60**	**36.43**	**38.65**	**38.90**	**42.39**	**0.420**	**-0.339**	**1.180**	**0.224**

BD = Bed-Days

In the overall ranking of ATC 4^th^ level groups (Table 3), the third-generation cephalosporins (ATC J01DD) and macrolides (J01FA) were the second and the third antibiotics with 6.90 DDD/100 BD (16.3%) and 5.67 DDD/100 BD (13.4%) in 2011, respectively. Group J01XD (imidazole derivatives) showed a significant increase in consumption from 2004 to 2011 (b = 0.055; IC95% 0.012–0.098; p = 0.020), due to the increase of metronidazole (+0.55 DDD/100 BD; +191.2%). The use of first-generation cephalosporins (J01DB) significantly increased during the 8 years considered (b = 0.035; IC95%: 0.006–0.063; p = 0.025). Groups J01CA (penicillins with extended spectrum) and J01EE (combinations of sulphonamides and trimethoprim) showed a statistically significant reduction (b = −0.156; IC95%: −0.254–−0.058; p = 0.008) (b = −0.068; IC95%: −0.120–−0.017; p = 0.018) (Table 3).

A total of 65 different active substances (ATC 5th level) out of the 175 antibiotics marketed in Italy, were used in the inpatient pediatric population. Amoxicillin/clavulanic acid was the most used drug, followed by ceftriaxone (11.4% of the total amount in 2011), ampicillin/sulbactam and clarithromycin (8.8% and 8.5% respectively). The frequency of newer and broad-spectrum drugs, such as daptomycin, was negligible.[S1 Table]

As far as the antibiotics prescribed by hospital physicians only after a patient-specific request to the hospital pharmacy (tigecycline, ertapenem, ceftaroline, telithromycin, quinupristin/daflopristin, teicoplanin, linezolid, daptomycin) are concerned, only 5 drugs out of the 8 antibiotics with restricted indications were used, and among them, linezolid and teicoplanin represented 0.6% and 1.4% of total consumption in 2011, respectively.[S1 Table]

### Pharmacovigilance analysis

Data from the Italian Spontaneous Reporting Database (VigiSegn) showed that only 27 spontaneous ADR reports associated to ATC J01 drugs were recorded in the pediatric wards of Emilia-Romagna hospitals between January 2004 and December 2011. Twenty-three cases involved children (3–13 years old), whereas 4 occurred in infants (≤2 years). Amoxicillin/clavulanic acid had the highest number of ADR reports (n = 7), and in 2 cases the ADR was recorded as serious.

## Discussion

In 2011, Italy ranked 8^th^ among European countries with the highest consumption of antibiotics in outpatient care.[10] A strong correlation between the extent of antibiotic consumption in outpatient and inpatient care has been shown,[36] however no national detailed data of hospital antibiotic use have been reported up to now. A previous survey aimed to describe the prevalence of antibiotic use in hospitalized children was carried out in a hospital in Rome, in June 2007.[33] The Italian National Report on drug use about outpatients showed that infants (< 4 years old) and elderly (> 55) people are more exposed to antibiotics than other age groups.[37]

The present is the first survey focused on the trend of antibiotic consumption in inpatient pediatric hospitals of a whole Italian Region, Emilia-Romagna, in 8 years.

This study showed a consumption of amoxicillin/clavulanic acid higher than that of amoxicillin plain. According to international guidelines and medical evidence,[5,38,39] plain amoxicillin should be preferred to the combination with the β-lactamase inhibitor; Emilia-Romagna regional guidelines [40] also suggest to use amoxicillin as the first choice drug to treat the most common infections in children (e.g. acute otitis media and streptococcal pharyngotonsillitis). Our findings are not aligned to national and international recommendations, probably owing to the increase of the amoxicillin-resistance in our Region.[41] These data are reflected in stable resistance rates for common pathogens in children, such as *Streptococcus pneumonia*, even if amoxicillin-resistant *Haemophilus influenzae* is going to increase over years.[41]

Although consumption of penicillins with extended spectrum (J01CA) decreased from 2004 to 2011 by 13.5% in the overall ranking, this class of antibiotics was the most consumed in neonatology, in line with the already cited guideline.[5,40] This evidence could be regarded as positive since penicillins represent the safest drugs especially in vulnerable persons such as infants.[42]

Third-generation cephalosporins were the second most used group in the overall ranking, in contrast with the guidelines that recommend to limit their use in children. However, it is to note that third-generation cephalosporins had only a modest not significant increase from 2004 to 2011, probably because of the actions in surveillance, education and policy development and implementation undertaken.[1,7–9,11,37]

The other antibiotics that could be regarded as second choice or to be reserved to individual critical patients (e.g. fluoroquinolones and carbapenems) had modest consumption and an almost stable time trend in the pediatric wards, in spite of their notable increase in other non pediatric wards of Emilia-Romagna hospitals reported by a previous study of ours.[34] This finding suggests that hospital antibiotic use in children tended to align to the guidelines for diagnosis and treatment of common infectious diseases, that recommend no prescription or at least delayed prescription for the most common upper respiratory tract infections.[5]

The use of drugs included in the patient-specific request list to restrict their consumption to the more serious cases (tigecycline, ertapenem, ceftaroline, telithromycin, quinupristin/daflopristin, teicoplanin, linezolid, daptomycin), was negligible. Among them, stands out linezolid, that is targeted to MRSA (*Methicillin-Resistant Staphylococcus Aureus*) infections and increased in consumption by 166.67% from 2004 to 2011. These data let us suppose a worrisome growth in glycopeptide-resistance or an inappropriate use of linezolid. From an economic standpoint, among the antibiotics, linezolid was the most expensive drug in 2011 (28.46 Euros/100 BD) representing alone 18.3% of the total amount. Therefore, linezolid use should be examined closely to keep the efficacy of the drug and in order to ensure the respect of labelled criteria. Finally, our results show that oral and parenteral administration routes were used in almost equal proportions. The relatively high consumption of parenteral preparations is partly due to the use of third-generation cephalosporins.

Safety of antibiotics is another debated issue. Our pharmacovigilance data seem to suggest a low frequency of antibiotics-associated ADRs in pediatric inpatients. By the way, a previous paper of ours showed a common and scarce attitude of healthcare providers to spontaneously report a possible ADR related to the use of a particular drug.[43] Though underreporting is a well known phenomenon and the voluntary nature of the reports creates inherent limitations in data interpretation. However, the majority of ADRs were not serious and were related to the most used drug amoxicillin/clavulanic acid.

Our study has some limitations affecting both drug utilization data and pharmacovigilance data. The analysis of hospital consumption data did not allow to evaluate the age- and gender-related differences, the appropriateness of antibiotic use and the presence of nosocomial infections because of the lack of patient and diagnosis information, as well as of microbiological data. Another limitation is represented by the use of defined daily dose as the tool to measure antibiotic consumption in the pediatric population. We are aware that DDD is intended for adult population and that pediatric age includes children who need different antibiotic doses depending on their body weight. However, the lack of patient information in the pharmacy records prevented us to use any other unit of measure.

The strength of this study is the large sample of the hospitals involved, the rigorous data collection method and a reliable estimate of exposure to antibiotics derived from detailed consumption registration in all hospitals.

## Conclusion

This study shows a modest increase of antibiotic utilization in pediatric inpatients, but the steadily increasing consumption in penicillins and β-lactamase inhibitors in association with a considerable decrease of plain penicillins, rises a serious concern. The clinical implications of the high use of third-generation cephalosporins and of linezolid should also be carefully evaluated.

Pharmacovigilance reports seems to suggest a safe use of antibiotics in the hospital setting of Emilia-Romagna, but this evidence could be biased by underreporting.

Our findings highlight the need to further investigate the reason for prescribing antibiotics in children inpatients, in order to improve health performance’s quality.

## Supporting Information

S1 Table Trend of antibiotic active substances (ATC 5th level) from 2004 to 2011 in pediatric inpatients in Emilia-Romagna hospitals. (DOCX)

## Abbreviations:

WHO: World Health Organization
ADRs: Adverse Drug Reactions
SRS: Spontaneous Reporting System
ATC: Anatomical Therapeutic Classification
DDD: Defined Daily Dose
BD: Bed-Days
UH: University Hospital
GH: General Hospital
MRSA: Methicillin-Resistant Staphylococcus Aureus
