# Supplementary material for: Use of Antibiotics in Pediatrics: 8-Years Survey in Italian Hospitals
Source: PLoS One. 2015 Sep 25;10(9):e0139097. doi: 10.1371/journal.pone.0139097 (PMC4584004; doi:10.1371/journal.pone.0139097)
Supplement: S1 Table — (DOCX) [file pone.0139097.s001.docx]

**S1 Table.** Trend of antibiotic active substances (ATC 5^th^ level) from 2004 to 2011 in pediatric inpatients in Emilia-Romagna hospitals. Drugs are ranked by decreasing consumption in 2011.

| **Rank** | **ATC** | **Active substance** | **DDD/100 BD** | | | | | | | | **Cumulative % (2011)** |
| --- | --- | --- | --- | --- | --- | --- | --- | --- | --- | --- | --- |
|  |  |  | **2004** | **2005** | **2006** | **2007** | **2008** | **2009** | **2010** | **2011** |  |
| 1 | J01CR02 | Amoxicillin and clavulanic acid | 10.31 | 11.76 | 11.65 | 11.43 | 10.48 | 10.44 | 10.22 | 11.15 | 26.30 |
| 2 | J01DD04 | Ceftriaxone | 4.48 | 4.80 | 4.50 | 4.33 | 4.02 | 4.67 | 4.66 | 4.84 | 37.72 |
| 3 | J01CR01 | Ampicillin and sulbactam | 0.47 | 1.74 | 1.98 | 2.03 | 2.36 | 3.00 | 3.37 | 3.71 | 46.47 |
| 4 | J01FA09 | Clarithromycin | 2.92 | 2.99 | 2.65 | 2.71 | 2.26 | 2.81 | 3.35 | 3.61 | 54.99 |
| 5 | J01CA04 | Amoxicillin | 3.87 | 3.91 | 3.29 | 3.28 | 2.93 | 2.88 | 2.56 | 2.72 | 61.41 |
| 6 | J01CA01 | Ampicillin | 1.90 | 2.12 | 1.94 | 2.36 | 2.08 | 2.19 | 2.25 | 2.33 | 66.90 |
| 7 | J01FA10 | Azithromycin | 0.79 | 1.39 | 1.48 | 1.74 | 1.41 | 1.75 | 1.62 | 1.80 | 71.15 |
| 8 | J01DD02 | Ceftazidime | 0.86 | 1.12 | 1.13 | 1.24 | 1.13 | 1.23 | 1.32 | 1.36 | 74.36 |
| 9 | J01GB06 | Amikacin | 0.93 | 1.08 | 1.49 | 1.24 | 1.28 | 1.04 | 1.22 | 1.16 | 77.09 |
| 10 | J01DC02 | Cefuroxime | 0.38 | 0.69 | 0.71 | 0.71 | 0.68 | 0.55 | 0.76 | 0.90 | 79.22 |
| 11 | J01DH02 | Meropenem | 0.59 | 0.80 | 0.96 | 0.71 | 0.81 | 0.93 | 0.52 | 0.89 | 81.32 |
| 12 | J01GB03 | Gentamicin | 0.76 | 0.56 | 0.60 | 0.58 | 0.80 | 0.81 | 0.73 | 0.87 | 83.37 |
| 13 | J01XD01 | Metronidazole | 0.28 | 0.45 | 0.72 | 0.48 | 0.61 | 0.66 | 0.63 | 0.83 | 85.33 |
| 14 | J01EE01 | Sulfamethoxazole and trimethoprim | 1.11 | 0.94 | 0.91 | 0.67 | 0.69 | 0.46 | 0.59 | 0.72 | 87.03 |
| 15 | J01XA02 | Teicoplanin | 0.66 | 0.59 | 0.59 | 0.68 | 0.58 | 0.65 | 0.61 | 0.59 | 88.42 |
| 16 | J01MA02 | Ciprofloxacin | 0.48 | 0.59 | 0.51 | 0.50 | 0.47 | 0.39 | 0.41 | 0.52 | 89.64 |
| 17 | J01EC02 | Sulfadiazine | 0.26 | 0.18 | 0.21 | 0.45 | 0.07 | 0.42 | 0.42 | 0.37 | 90.52 |
| 18 | J01CR05 | Piperacillin and tazobactam | 0.12 | 0.11 | 0.21 | 0.23 | 0.27 | 0.29 | 0.27 | 0.36 | 91.37 |
| 19 | J01DD08 | Cefixime | 0.50 | 0.57 | 0.57 | 0.63 | 0.60 | 0.55 | 0.38 | 0.34 | 92.17 |
| 20 | J01DB04 | Cefazolin | 0.11 | 0.13 | 0.31 | 0.35 | 0.38 | 0.34 | 0.38 | 0.33 | 92.95 |
| 21 | J01XA01 | Vancomycin | 0.15 | 0.19 | 0.21 | 0.21 | 0.22 | 0.33 | 0.28 | 0.32 | 93.70 |
| 22 | J01GB01 | Tobramycin | 0.82 | 0.71 | 0.64 | 0.80 | 0.17 | 0.24 | 0.37 | 0.28 | 94.36 |
| 23 | J01MA12 | Levofloxacin | 0.21 | 0.12 | 0.18 | 0.23 | 0.23 | 0.15 | 0.23 | 0.27 | 95.00 |
| 24 | J01FA01 | Erythromycin | 0.49 | 0.37 | 0.35 | 0.34 | 0.36 | 0.34 | 0.34 | 0.26 | 95.61 |
| 25 | J01CF04 | Oxacillin | 0.16 | 0.18 | 0.11 | 0.20 | 0.22 | 0.21 | 0.22 | 0.25 | 96.20 |
| 26 | J01XX08 | Linezolid | 0.09 | 0.08 | 0.02 | 0.03 | 0.07 | 0.12 | 0.18 | 0.24 | 96.77 |
| 27 | J01DD14 | Ceftibuten | 0.09 | 0.03 | 0.02 | 0.02 | 0.00 | 0.02 | 0.16 | 0.23 | 97.31 |
| 28 | J01DC04 | Cefaclor | 0.65 | 0.57 | 0.54 | 0.43 | 0.33 | 0.18 | 0.15 | 0.18 | 97.74 |
| 29 | J01XB01 | Colistin | 0.20 | 0.39 | 0.29 | 0.30 | 0.22 | 0.20 | 0.08 | 0.16 | 98.11 |
| 30 | J01DC01 | Cefoxitin | 0.09 | 0.12 | 0.11 | 0.15 | 0.16 | 0.10 | 0.16 | 0.15 | 98.47 |
| 31 | J01FF01 | Clindamycin | 0.03 | 0.06 | 0.04 | 0.04 | 0.05 | 0.08 | 0.07 | 0.13 | 98.77 |
| 32 | J01DD01 | Cefotaxime | 0.06 | 0.08 | 0.08 | 0.08 | 0.08 | 0.13 | 0.07 | 0.12 | 99.06 |
| 33 | J01GB07 | Netilmicin | 0.15 | 0.1 | 0.11 | 0.15 | 0.09 | 0.08 | 0.05 | 0.06 | 99.20 |
| 34 | J01CE02 | Phenoxymethylpenicillin | - | - | - | - | - | 0.06 | 0.06 | 0.06 | 99.34 |
| 35 | J01DH51 | Imipenem and cilastatine | 0.06 | 0.1 | 0.04 | 0.03 | 0.02 | 0.04 | 0.01 | 0.04 | 99.43 |
| 36 | J01DE01 | Cefepime | 0.09 | 0.06 | 0.03 | - | - | 0.01 | 0.04 | 0.03 | 99.50 |
| 37 | J01DF01 | Aztreonam | 0.05 | 0.06 | <0.01 | 0.05 | 0.06 | 0.03 | <0.01 | 0.03 | 99.58 |
| 38 | J01CE08 | Benzathine benzylpenicillin | 0.02 | 0.01 | 0.02 | 0.01 | 0.02 | 0.02 | 0.01 | 0.03 | 99.65 |
| 39 | J01BA01 | Chloramphenicol | 0.04 | 0.10 | 0.07 | 0.06 | 0.04 | 0.04 | 0.03 | 0.02 | 99.69 |
| 40 | J01XE01 | Nitrofurantoin | 0.01 | 0.03 | <0.01 | <0.01 | 0.01 | <0.01 | 0.01 | 0.02 | 99.74 |
| 41 | J01MB04 | Pipemidic Acid | - | - | - | - | - | - | - | 0.02 | 99.79 |
| 42 | J01XX09 | Daptomycin | - | - | - | - | - | - | 0.03 | 0.02 | 99.83 |
| 43 | J01CA12 | Piperacillin | 0.08 | 0.08 | 0.07 | 0.03 | 0.02 | 0.04 | 0.03 | 0.01 | 99.86 |
| 44 | J01AA02 | Doxycycline | 0.04 | 0.02 | 0.03 | 0.02 | 0.04 | 0.05 | 0.02 | 0.01 | 99.88 |
| 45 | J01CE01 | Benzylpenicillin | 0.03 | 0.01 | 0.01 | <0.01 | 0.02 | 0.01 | <0.01 | 0.01 | 99.91 |
| 46 | J01XX01 | Fosfomycin | 0.02 | 0.03 | 0.01 | <0.01 | 0.01 | 0.01 | 0.03 | 0.01 | 99.93 |
| 47 | J01MA14 | Moxifloxacin | 0.01 | 0.01 | 0.03 | 0.01 | <0.01 | 0.02 | - | 0.01 | 99.95 |
| 48 | J01AA12 | Tigecycline | - | - | - | <0.01 | 0.03 | 0.03 | <0.01 | 0.01 | 99.98 |
| 49 | J01DD13 | Cefpodoxime | - | <0.01 | <0.01 | <0.01 | - | - | <0.01 | 0.01 | 100.00 |
| 50 | J01FA07 | Josamycin | 0.05 | 0.02 | 0.02 | 0 | - | <0.01 | - | <0.01 | 100.00 |
| 51 | J01CR04 | Sultamicillin | - | - | - | - | - | <0.01 | - | 0 | 100.00 |
| 52 | J01DC06 | Cefonicid | 0.05 | 0.06 | 0.06 | - | - | - | - | - | 100.00 |
| 53 | J01BA02 | Thiamphenicol | 0.02 | 0.03 | 0.02 | 0.01 | 0.01 | 0.01 | <0.01 | - | 100.00 |
| 54 | J01CE30 | Combinations | 0.02 | - | - | - | - | - | - | - | 100.00 |
| 55 | J01FA02 | Spiramycin | 0.01 | 0.02 | 0.01 | 0.03 | <0.01 | 0.01 | <0.01 | - | 100.00 |
| 56 | J01FA12 | Rokitamycin | 0.01 | - | 0.01 | <0.01 | - | - | - | - | 100.00 |
| 57 | J01CR03 | Ticarcillin and clavulanic acid | <0.01 | 0.02 | 0.01 | 0.01 | 0.01 | 0.01 | - | - | 100.00 |
| 58 | J01DC05 | Cefotetan | <0.01 | - | - | - | - | - | - |  | 100.00 |
| 59 | J01GA01 | Streptomycin | <0.01 | 0.01 | - | - | <0.01 | <0.01 | - | - | 100.00 |
| 60 | J01MA06 | Norfloxacin | <0.01 | - | - | - | - | - | - | - | 100.00 |
| 61 | J01DB01 | Cefalexin | - | <0.01 | - | 0.01 | - | - | - | - | 100.00 |
| 62 | J01DC03 | Cefamandole | - | <0.01 | - | - | - | - | - | - | 100.00 |
| 63 | J01DD07 | Ceftizoxime | - | <0.01 | <0.01 | - | - | - | - | - | 100.00 |
| 64 | J01FF02 | Lincomycin | - | <0.01 | - | - | <0.01 | - | - | - | 100.00 |
| 65 | J01FG02 | Quinupristin/Dalfopristin | - | - | - | - | - | <0.01 | - | - | 100.00 |
